# Detection of impaired renal allograft function in paediatric and young adult patients using arterial spin labelling MRI (ASL-MRI)

**DOI:** 10.1038/s41598-022-04794-y

**Published:** 2022-01-17

**Authors:** Tijana Radovic, Milica M. Jankovic, Ruza Stevic, Brankica Spasojevic, Mirjana Cvetkovic, Polina Pavicevic, Ivana Gojkovic, Mirjana Kostic

**Affiliations:** 1 Department of Radiology, University Children’s Hospital, Belgrade, Serbia; 2 Department of Signals and Systems, School of Electrical Engineering, University of Belgrade, Belgrade, Serbia; 3 School of Medicine, University of Belgrade, Belgrade, Serbia; 4 Department of Radiology, Clinical Centre of Serbia, Belgrade, Serbia; 5 Department of Nephrology, Dialysis and Transplantation, University Children’s Hospital, Belgrade, Serbia

**Keywords:** Biomedical engineering, Nephrology, Urology

## Abstract

The study aimed to discriminate renal allografts with impaired function by measuring cortical renal blood flow (cRBF) using magnetic resonance imaging arterial spin labelling (ASL-MRI) in paediatric and young adult patients. We included 18 subjects and performed ASL-MRI on 1.5 T MRI to calculate cRBF on parameter maps. cRBF was correlated to calculated glomerular filtration rate (GFR) and compared between patient groups with good (GFR ≥ 60 mL/min/1.73 m^2^) and impaired allograft function (GFR < 60 mL/min/1.73 m^2^). Mean cRBF in patients with good allograft function was significantly higher than in patients with impaired allograft function (219.89 ± 57.24 mL/min/100 g vs. 146.22 ± 41.84 mL/min/100 g, *p* < 0.008), showing a highly significant correlation with GFR in all subjects (r = 0.75, *p* < 0.0001). Also, the diffusion-weighted imaging (DWI-MRI) apparent diffusion coefficient (ADC) and Doppler measurements of peak-systolic and end-diastolic velocities and the resistive index (PS, ED, RI) were performed and both methods showed no significant difference between groups. ADC implied no correlation with GFR (r = 0.198, *p* = 0.464), while PS indicated moderate correlation to GFR (r = 0.48, *p* < 0.05), and PS and ED moderate correlation to cRBF (r = 0.58, *p* < 0.05, r = 0.56, *p* < 0.05, respectively). Cortical perfusion as non-invasively measured by ASL-MRI differs between patients with good and impaired allograft function and correlates significantly with its function.

## Introduction

Kidney transplantation is a gold standard in treating patients with end-stage renal disease (ESRD). However, numerous posttransplant complications are frequent both in the early and late postoperative stages. Understanding the pathogenic mechanism of allograft injury and its on-time identification is crucial to preventing irreversible nephron loss and graft failure^1^. Hypoxia and insufficient nutrient delivery, which lead to tubular atrophy, capillary injury, and interstitial fibrosis, have been suggested as common causes of allograft injury^2,3^.

The chief parameter which regulates renal function is its blood perfusion. Perfusion is defined as blood flow at the level of the tissue capillary bed and determines the delivery of nutrients and oxygen to the tissue^4^. Renal perfusion is also a key determinant of glomerular filtration, therefore, a central measure of renal function monitoring. The development of a non-invasive and reliable method for renal perfusion estimation that would reflect glomerular filtration rate (GFR), along with providing precise allograft morphological assessment, would significantly improve on-time identification of potential allograft injury^5^.

Most procedures used so far for estimating renal allograft function have been considered either invasive or unreliable. Renal biopsy and dynamic scintigraphy, as gold standards, are both considered invasive with serious adverse events^6,7^. Further, ultrasonography is operator-dependent, often insensitive, and nonspecific. Serum creatinine levels are also used to determine renal function by estimating GFR; however, this method is insensitive to small but potentially significant function loss and may reflect it in the late stage of allograft function deterioration^8^.

MRI is a reliable tool for allograft anatomy and potential complications assessment at any posttransplant stage^9^. In addition, MRI can yield functional parameters as well, either using diffusion-weighted imaging (DWI), which provides an apparent diffusion coefficient (ADC), or a contrast-enhanced MRI study^10,11^. However, conventional MRI has its limitations. ADC values that quantify molecular diffusion in tissue are influenced by pure water diffusion, microvascular perfusion, and tubular flow and thus cannot reflect tissue perfusion per se^12^. Moreover, the use of an MRI contrast agent in renal allograft patients with poor kidney function is considered hazardous due to the potential risk of developing nephrogenic systemic fibrosis (NSF)^13^.

In recent years, arterial spin labelling (ASL) MRI has been applied in various anatomical regions to estimate tissue perfusion. This MRI technique uses arterial blood water as a freely diffusible natural tracer to track and quantify tissue perfusion^3,4,14–16^. It is considered completely safe and non-invasive, with high potential in assessing renal allograft function. In an ASL experiment, during image acquisition in the tissue of interest, two sets of images are obtained: first, a tag image that alters the longitudinal magnetization of arterial blood before it flows into the imaging plane, and second, a control image, without altering the magnetization of the inflowing blood^3,4^. By subtracting these two images, a perfusion-weighted image (PWI) is acquired. After feeding the PWI into a mathematical model that describes the relationship between the signal difference and the actual blood perfusion, the main result that is obtained is a quantitative perfusion map in relevant units (mL/100 g of tissue/min)^3–5^.

Initial research results reported the robustness and reliability of ASL-MRI in this clinical scenario^14–19^. However, the studies analysing the utility of ASL-MRI perfusion of renal allografts were mostly done in the adult population, and investigations including paediatric populations are greatly lacking^3–5,14–20^, with a few exceptions such as one study evaluating the native kidney in paediatric patients but not allografts^21^. In addition, we used a FAIR labelling scheme and 3D imaging that was considered more reliable and sensitive for abdominal use^3–5,14–19^. Therefore, the aim of this study was to analyse the impact of ASL-MRI perfusion on the functional evaluation of renal allografts in paediatric patients using a FAIR labelling scheme and 3D imaging module with background suppression.

## Methods

This prospective study was approved by the University Children’s Hospital Ethics Committee (application numbers 14/303/22/08/2018) and informed consent was obtained from all participants and their legal guardians, if necessary.

### Patients

After careful triage to determine the patients' eligibility for the study, 21 patients met the criteria for stable allograft function. They were all examined in our hospital between November 2018 and December 2019. Three patients were excluded due to poor cooperation and motion artefacts, leaving us with a sample size of 18 paediatric and young adult kidney transplant patients (10 male, median age 16 yrs.). In this final sample, one patient was 29 years old and was still a longstanding patient of our Paediatric Hospital at the time of the study. After thorough clinical evaluation, we included this participant with abiding chronic kidney disease that had influenced their growth and development, causing a significant lag in their bone age before transplantation, which took place in their very early adulthood. The data acquired in this patient showed no outlier characteristics.

All patients were recruited during routine clinical check-ups or their hospital stays and were advised to refrain from food and water intake for at least 4 h before their check-up, therefore, prior to the study day. Ten of them received an organ from a related living donor, while 8 received cadaveric renal allografts. The time interval between transplantation and the MRI exam varied from 2 weeks to 187 months, with a median of 74 months. At the time of the MR examination, all patients from the study population showed stable allograft function, without clinical signs of acute rejection, as well as in the subsequent six months, which was the most important inclusion criterion. Patients’ demographic data are shown in detail in Table 1.

**Table 1:** Clinical data of the patient population included in this study.

Patient no.	Gender	Age (y)	BMI (kg/m^2^)	Time-interval since transplantation (months)	Type of donor	Primary disease	HTA	Plasma creatinine (µmol/L)	GFR (mL/min/1.73 m^2^)	ASL (mL/min/100 g)
1	M	5	17.72	1	Living	CAKUT	No	73	55.56	136
2	F	18	17.8	13	Cadaveric	CAKUT	No	119	46.25	178
3	F	18	19.04	1	Cadaveric	CAKUT	No	69	66.82	160
4	F	19	25.56	66	Cadaveric	Nephronophthisis	Yes	107	76.46	210
5	M	19	25.4	1	Living	Nephronophthisis	No	144	36.23	85
6	M	29	20.2	83	Cadaveric	Nephrotic syndrome	Yes	108	65.25	249
7	M	16	19.6	4	Cadaveric	Nephronophthisis	Yes	146	77.67	211
8	M	15	28.27	45	Living	CAKUT	Yes	93	59.1	164
9	F	20	20.04	187	Living	Nephronophthisis	Yes	102	50.99	141
10	F	15	21.8	79	Cadaveric	Wilms tumour	No	90	64	109
11	F	9	15.68	82	Living	Congenital nephrotic syndrome	Yes	81	72.85	261
12	F	13	23.32	108	Living	Congenital nephrotic syndrome	No	117	57.09	204
13	M	13	16.55	112	Living	Congenital nephrotic syndrome	Yes	86	80.27	243
14	M	13	22.02	73	Living	Nephronophthisis	Yes	81	58.03	114
15	F	13	37.29	75	Living	Nephronophthisis	Yes	108	32.08	100
16	M	14	18.67	12	Cadaveric	CAKUT	No	68	93.39	303
17	M	19	21.63	161	Living	CAKUT	Yes	171	66.06	233
18	M	21	32.98	109	Cadaveric	CAKUT	Yes	206	41.58	194

M: male; F: female; BMI: body mass index; HTA: hypertension; GFR: glomerular filtration rate; ASL: arterial spin labelling perfusion; CAKUT: congenital anomalies of the kidney and urinary tract.

According to allograft function measured by clinical parameters (GFR), patients were assigned to two groups: group I comprised patients with good renal function (9 patients, GFR ≥ 60 mL/min/1.73 m^2^) and group II patients with impaired renal function (9 patients, GFR < 60 mL/min/1.73 m^2^). The threshold of 60 mL/min/1.73 m^2^ was selected because chronic kidney disease is defined as kidney damage or a GFR below this value for more than three months^22^. All blood samples and 24-h collected urine samples were obtained prior to the MRI exam, and creatinine levels in blood and urine were measured to calculate GFR. Below is the equation used to determine GFR, typically recorded in volume per time (e.g., mL/min)^23^:

$${\text{GFR}} = \left[ {{\text{UrineCr}}\;\left( {\text{mL/mg}} \right)} \right]*{\text{urine}}\;{\text{flow}}\;\left( {\text{mL/min}} \right){/}\left[ {{\text{PlasmaCr}}\;\left( {\text{mL/mg}} \right)} \right].$$

### Ultrasound measurements

Doppler evaluation of allograft perfusion was performed on the same day as the MRI examination, measuring the peak-systolic (PS) and end-diastolic (ED) velocity peripherally in interlobular cortical branches, and the resistive index (RI) was calculated according to the following equation^24^:

$${\text{RI}} = \left[ {{\text{PS}} - {\text{ED}}} \right]/{\text{PS}}.$$

### MR imaging

MR examinations were performed on a 1.5-T whole-body MRI scanner (Magnetom Aera, Siemens AG, Healthcare Sector, Erlangen, Germany). Patients were examined in the supine position. Image acquisition was done during free breathing using a six-channel body matrix array coil placed over the abdominal and/or pelvic region in combination with the spine matrix coil built into the scanner table. In all subjects, standard coronal T2-weighted HASTE (Half-Fourier Acquired Single Shot Turbo Spin Echo) and axial T1-weighted 3D volumetric VIBE (volume interpolated breath-hold) sequences were acquired for anatomical evaluation of the transplanted kidney. A monoexponential model of diffusion-weighted imaging (DWI) MRI was acquired by the EPI-SE sequence type in the axial plane for complete allograft coverage during free respiration with b values of 50/400/600 s/mm^2^ and automated ADC parameter maps were generated by the MR systems. The total scanning time was 2.35 min. For the quantitative ASL assessment of transplanted kidney perfusion, flow-sensitive alternating inversion recovery true–fast imaging with steady-state precession (FAIR True-FISP) and multi-shot 3D gradient and spin-echo (3D-GRASE) imaging module with background suppression ASL technique was performed^25–30^. The images were acquired in an oblique-sagittal orientation to avoid inclusion of the aorta or the pelvic arteries in the acquisition window. An adiabatic frequency offset correction inversion pulse placed in line with the imaging slab was used, positioned to exclude the feeding arteries^25^. The selective inversion slab with non- selective and selective pulses was dependent on the imaging stack thickness (56 mm). For clearly defining the bolus duration, Q2TIPS was applied to cover the feeding arteries^26–30^. Imaging parameters were as follows: number of segments-3 (multi-shot), field of view 360 × 360 mm, TE/TR 20/6000 ms, Flip angle 180, without parallel imaging, voxel size 2.8 × 2.8 × 6 mm, slice thickness 6 mm, number of slices 8, reconstruction matrix 128 × 128, measurement 6 (paired tagged and control images). To allow for sufficient labelled blood to perfuse into the tissue, an inversion pulse delay time (TI) of 2000 ms was used^29^. The total scan time for the FAIR True-FISP sequence was 3.42 min. An additional proton density image without inversion was acquired for the determination of the equilibrium magnetization M0, which is necessary for perfusion quantification, following the field of view, slice orientation, slice thickness, and reconstruction matrix to match FAIR ASL. The total scan time was 1.36 min. A T1 value was set to 1.156 s, as described in the literature for renal allografts^30,31^.

All performed sequences are presented in detail in Table 2.

**Table 2:** MRI parameters of the used sequences.

Sequence type	T2 HASTE	3D T1 VIBE	DWI	ASL-MRI	PDW
Sequence type	SSh FSE	Spoiled 3D GRE	EPI-SE	FAIR-True FISP	TSE
Slice orientation	Coronal	Axial	Axial	Para-sagittal	Para-sagittal
FOV (mm × mm)	350 × 350	380 × 308	350 × 282	360 × 360	360 × 360
TR/TE (ms)	2000/92	4.74/2.08	2000/53	6000/20	4000/35
TI (ms)	NA	NA	NA	2000	NA
Flip Angle	180	10	NA	180	150
b values (s/mm^2^)	NA	NA	50/400/600	NA	NA
Slice thickness (mm)	5	3	6	6	6
Interslice gap (mm)	1	0.3	0	1.2	1.2
Number of slices	32	30	35	8	8
Matrix	256 × 256	166 × 256	108 × 134	128 × 128	128 × 128
Spatial resolution (mm × mm × mm)	1.4 × 1.4 × 5	1.5 × 1.5 × 3	2 × 2 × 6	2.8 × 2.8 × 6	2.8 × 2.8 × 6
Parallel imaging	GRAPPA	CAIPIRINHA	GRAPPA	-	GRAPPA
Fat suppression	None	SPAIR	SPAIR	Fat Sat	None
Respiratory control	Free-breathing	Breath-hold	Free-breathing	Free-Breathing	Free-breathing
Bandwidth (Hz/px)	698	350	2332	3004	260
Acquisition time (min)	2.30	0.12	2.35	3.42	1.36

HASTE—Half-Fourier Single-Shot Turbo Spin-Echo; VIBE—Volumetric Interpolated Breath-hold Examination; DWI—Diffusion-Weighted Imaging; ASL—Arterial Spin Labeling; PDW- Proton Density-Weighted; SSh FSE—Single-Shot Fast Spin-Echo; GRE—Gradient Echo; EPI SE—Echo-Planar Imaging Spin-Echo; FAIR True-FISP—Flow-sensitive Alternating Inversion Recovery True-Fast Imaging with Steady-State Precision; FOV—Field of View; TR—Repetition Time; TE—Echo Time; TI—Inversion Time; GRAPPA—Generalized Autocalibrating Partial Parallel Acquisition; CAIPIRINHA—Controlled Aliasing in Parallel Imaging Results in Higher Acceleration; GRASE- Gradient and Spin Echo; SPAIR—Spectral Attenuated Inversion Recovery; Fat Sat—Fat Saturation.

### Image analysis

For the ASL-MRI parametric image analysis, in-house software was developed based on the Labview environment version 2018 (National Instruments, Texas, Austin) and an additional NI Vision Development module. Input images for the software were tag and control images, as well as the corresponding structural proton density image (PDW). All images were registered and analysed qualitatively for motion artefacts and images that were not aligned or uncooperative participants were discarded. The following equation was used for the pixel-based calculation of cortical perfusion, visually presented to the user by the parametric map^3,4^:

$$f = \frac{\lambda \varDelta M}{{2TI \cdot M_{o} }} \cdot {\text{e}}\left| {\frac{TI}{{T_{1} }}} \right|$$

The calculation is based on the difference in longitudinal magnetization (ΔM) between ASL FAIR acquisitions with tag and control images. M0 represents the tissue equilibrium magnetization per unit mass measured by a separate sequence (PDW image) as described above. ƒ is the perfusion rate in cRBF and λ the blood–tissue water partition coefficient, which is assumed to have a constant value of 80 mL/100 g. For regional perfusion analysis, the cortex of the renal allograft was defined on coloured perfusion maps using in-house software, and a hand-drawn region of interest (ROI) was defined by two experienced examiners with the result of an automatically calculated mean value and standard deviation. In addition, relative perfusion-weighted signal (PWS) for quality evaluation was calculated according to the equation^27,34^:

$$PWS = \frac{\varDelta M}{{M_{o} }} \cdot 100 \left( \% \right).$$

**Figure 1:**
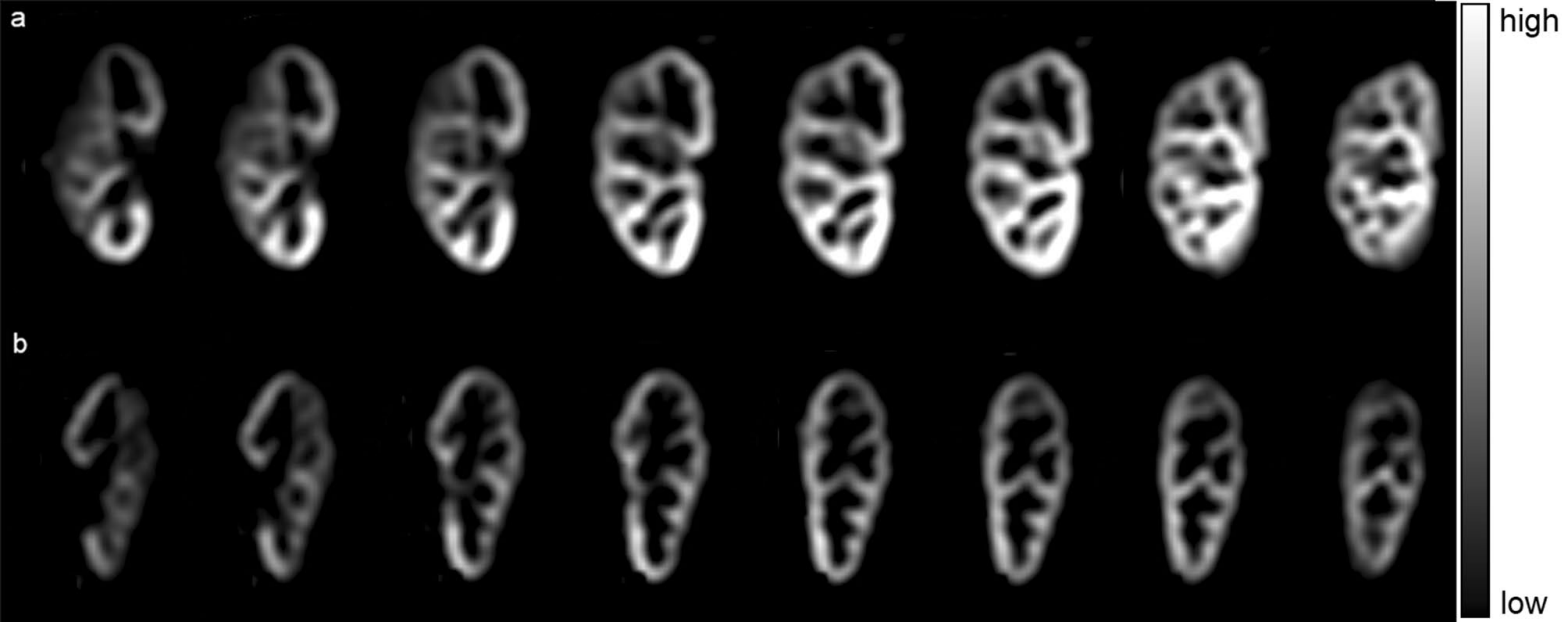
Multi-slice perfusion weighted images (PWI) in two representative patients with: (**a**) good allograft function (GFR ≥ 60 ml/min/100 g) and (**b**) impaired allograft function (GFR < 60 ml/min/100 g).

Since we have used 3D data with 8 slices, there were 8 PWI/RBF maps generated in each participant, which were qualitatively and quantitatively assessed by the author experienced in abdominal and urogenital imaging in order to choose the best slice for final quantification (Fig. 1). In each participant, maps with significant noise were excluded (mostly generated from slices at the very beginning or end of the imaging stack, mainly due to partial volume effect with the nearby structures). The middle slice (the 4th or 5th one, depending on the exact position of the imaging stack) was the most representative one, and these were of good quality in all participants.

**Figure 2:**
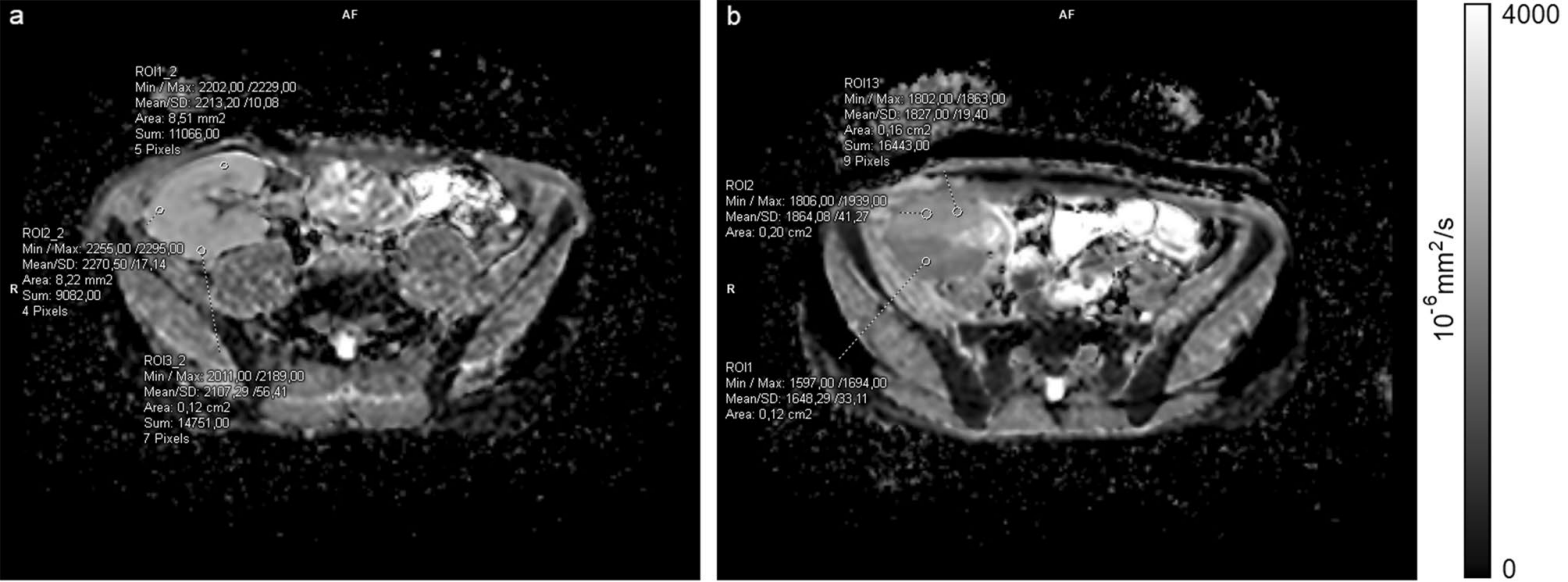
Axial ADC maps with representative circular regions of interest (ROI) placed in the renal cortex in: (**a**) patient with GFR ≥ 60 mL/min/1.73 m^2^ and (**b**) patient with GFR ≥ 60 mL/min/1.73 m^2^ (notice the difference in ADC values of anterior and posterior aspect of the kidney, with moderate diffusion restriction in posterior aspect after intraoperative vascular incident; this DWI appearance resolved after few months from transplantation).

For diffusion quantification, the acquired ADC maps were analysed on custom clinical workstations and ADC was measured by placing multiple ROIs in the renal cortex in all acquired slices (3 ROIs per slice) to calculate the mean cortical value (Fig. 2). For a precise ROI placement, cortico-medullary differentiation was accessed on the VIBE sequence and a 3D reference point was used for navigation.

### Statistics

All statistical analyses were calculated using the Statistical Package for Social Sciences (SPSS v. 25.0, IBM, Chicago, IL). For analysing the difference in median time from transplantation and the difference in median age, the Mann–Whitney test was employed. Independent sample *t* test with the Bonferroni correction to account for multiple testing was used to compare differences in the mean GFR, cRBF, and ADC values, mean peak-systolic (PS), end-diastolic (ED) velocities, and resistive indices (RI) between two patient groups. After applying the Bonferroni correction, *p* < 0.008 was considered statistically significant. Also, all parameters were correlated to the GFR value in the whole patient group using Pearson’s correlation coefficient to determine their connection to allograft function. The receiver operating characteristic (ROC) curve and binary logistic regression analyses were performed to assess the diagnostic efficiency of cRBF, ADC, PS, ED, and RI in distinguishing allografts with impaired function from those with normal function. Interobserver reproducibility for ASL cortical measurements between two readers was evaluated using an intraclass correlation coefficient.

### Ethical approval

All procedures performed in the studies involving human participants were in accordance with the ethical standards of the institutional and/or national research committee and the 1964 Helsinki declaration and its later amendments or comparable ethical standards. This prospective study was approved by the University Children’s Hospital Ethics Committee (application numbers 14/303/22/08/2018).

### Consent to participate

Written informed consent was obtained from all participants and their guardians if necessary.

### Consent for publication

All of the authors have approved the content of this paper and have agreed to the Scientific Reports’ submission policies as well as the responsible authorities at the institute where the research has been carried out.

## Results

MR acquisition was completed successfully in all patients. All the studies generated overall good-quality maps. Because all participants had kidney transplants located in the pelvic region, their position was not significantly affected by respiratory motion during image acquisition^3^. Intestinal peristalsis was found to be a possible source of motion artefacts, which was resolved along with other important clinical factors by organising patients to come early in the morning fasted from food and water from the night before. To allow for quality assessment, PWS was calculated with a mean value of 3.43 ± 1.43%, which corresponds to the literature^27,34^, with no significant difference between patient groups (*p* = 0.101). Since 3D acquisition was performed, cRBF values of the whole kidney measured in all slices were compared to a single representative middle slice and the acquired results showed a highly significant correlation between the two values (r = 0.82, *p* = 0.00004) with no statistically significant difference in the mean value in two different approaches (whole kidney vs single representative slice mean values 173.78 ± 53.29 and 183 ± 61.66 ml/min/100 g, respectively, *p* = 0.319).

According to their GFR values (group I with GFR ≥ 60 mL/min/1.73 m^2^ and group II with GFR < 60 mL/min/1.73 m^2^), 9 patients were included in each group. The median age in group I was 16 yrs., and in group II 15 yrs., with no statistically significant difference in age (*p* = 0.722). The median time interval from transplantation in group I was 79 months and in group II 73 months, without statistically significant difference (*p* = 0.895). Eleven of 18 patients had controlled hypertension with two or three antihypertensive medicaments (6 from group I and 5 from group II; 2 from each group received ACE inhibitors).

**Figure 3:**
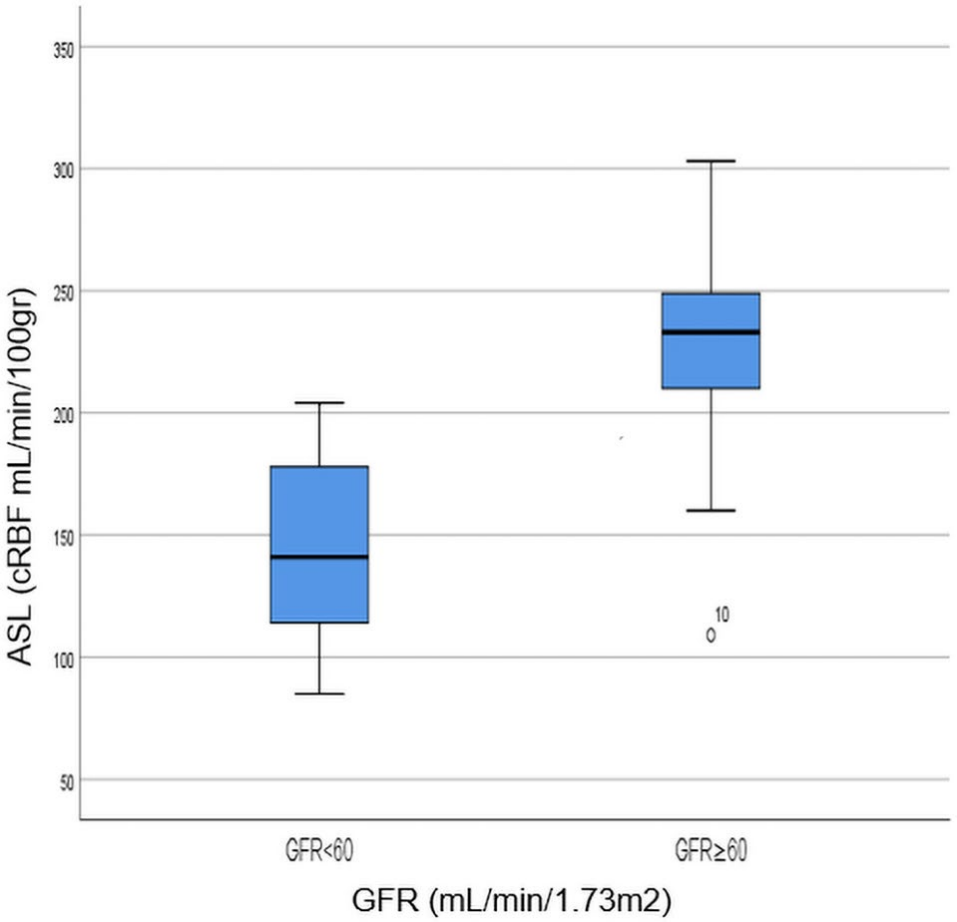
Box plot showing a comparison of perfusion values in patients with GFR < 60 and GFR ≥ 60 mL/min/1.73 m^2^; an outlier from group 2 (GFR ≥ 60 mL/min/1.73 m^2^) falling out of the boxplot corresponds to the participant number 10 in Table 1.

**Figure 4:**
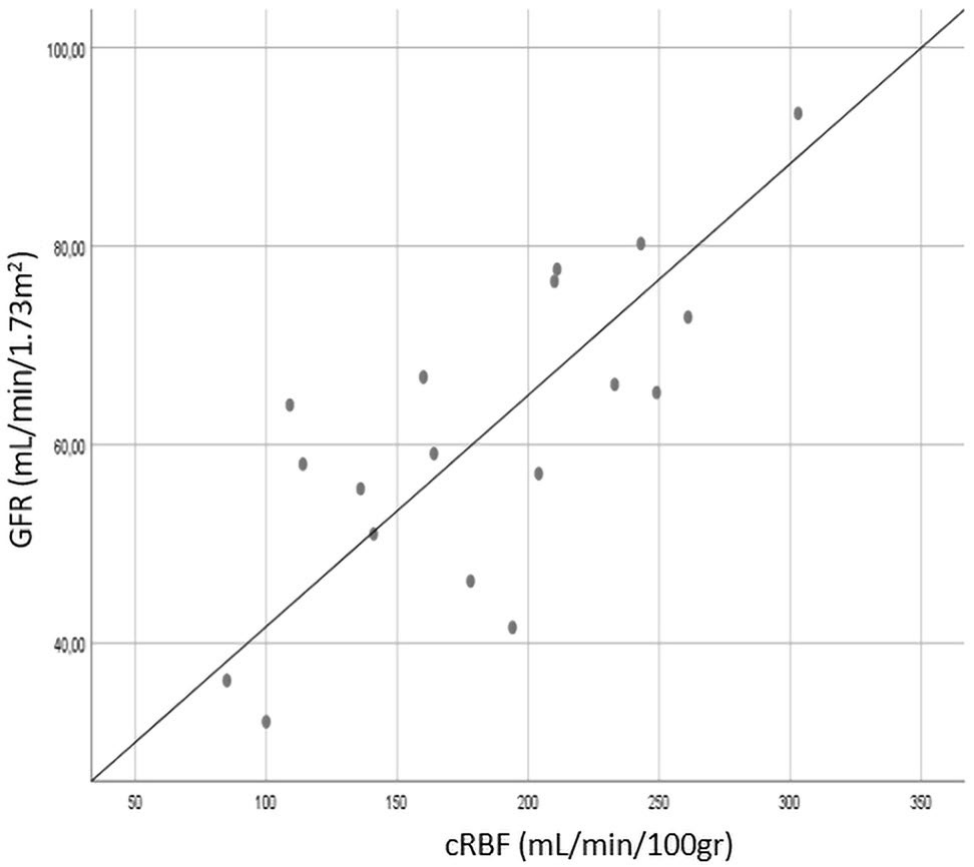
Scatter plot showing a positive correlation coefficient between cRBF and GFR; r = 0.75, *p* < 0.0001.

**Figure 5:**
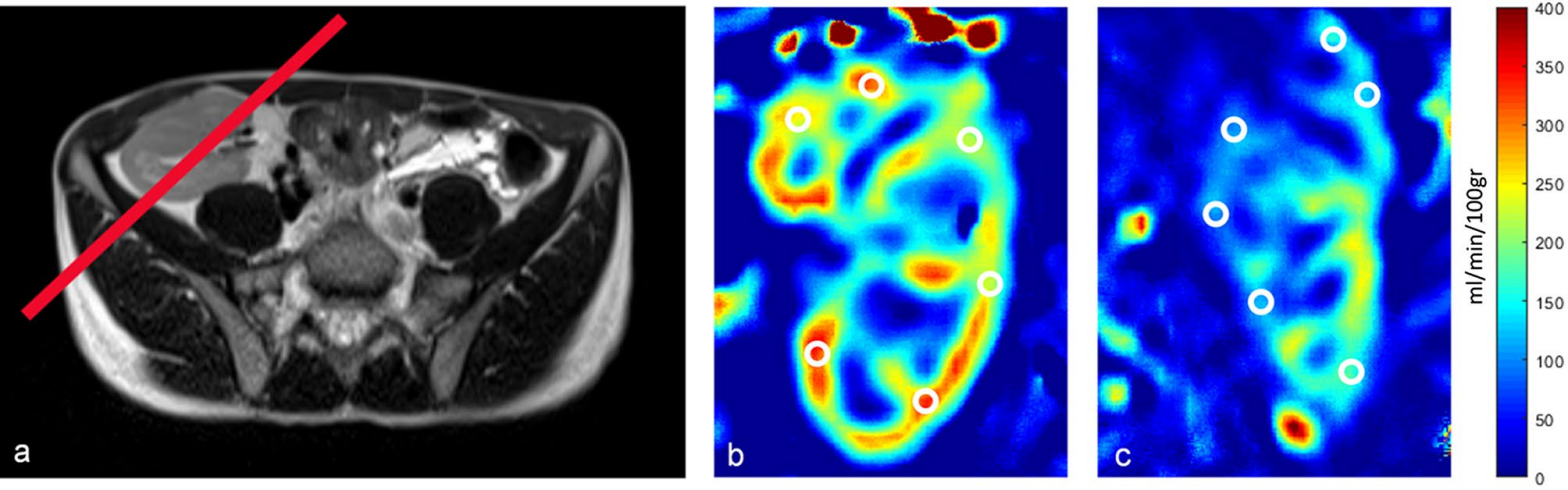
(**a**) Positioning of the middle slice; (**b**) Quantitative Perfusion Map representing cRBF (ml/min/100 g) imaged in a patient with good allograft function (GFR ≥ 60 ml/min/100 g) and (**c**) Quantitative Perfusion Map representing cRBF (ml/min/100 g) imaged in a patient with impaired allograft function (GFR < 60 ml/min/100 g).

The mean GFR value in group I was 73.64 ± 9.53 mL/min/1.73 m^2^ and in group II 48.54 ± 10.04 mL/min/1.73 m^2^, showing a statistically significant difference, as expected (*p* < 0.0001). The mean cRBF value of ASL perfusion in group I was 219.89 ± 57.24 ml/min/100 g, while in group II it was 146.22 ± 41.84 ml/min/100 g, suggesting significantly higher values in group I compared to group II (*p* = 0.0066, Fig. 3). Furthermore, ASL perfusion showed a highly significant positive correlation with GFR values (r = 0.75, *p* < 0.0001, Fig. 4). The middle slice position and qualitative and quantitative differences in the perfusion maps between patients with good and impaired function along with ROI placement are presented in Fig. 5. The interobserver variability as assessed by the intraclass correlation coefficient for ASL was 0.96 (95% confidence interval [CI], 0.92–0.98).

For further statistical analysis, the study group was divided into two groups according to the donor type (living donor LD and cadaveric donor CD). The mean GFR value was then calculated as 56.82 ± 14.79 mL/min/1.73 m^2^ in patients with an LD allograft and 66.42 ± 16.84 mL/min/1.73 m^2^ in patients with a CD allograft, suggesting no statistically significant difference in the values (*p* = 0.225). Also, the mean cRBF reflected this finding, showing no statistically significant difference in mean values between types of allografts (in LD 168.10 ± 63.24 mL/min/100 g, in CD 201.75 ± 58.07 mL/min/100 g, *p* = 0.262).

In the cortical DWI analysis comparing ADC values with GFR values, the mean ADC value in group I was 1.97 ± 0.06 × 10^−3^ mm^2^/s, while the mean value in group II was 1.91 ± 0.07 × 10^−3^ mm^2^/s, with no statistically significant difference between groups (*p* = 0.585) and without statistically significant correlation of the overall cortical ADC value with the GFR (r = 0.198, *p* = 0.464).

Comparison of the measured Doppler cortical perfusion values revealed the mean PS, ED, and RI were 21.33 ± 4.28 cm/s, 7.94 ± 1.38 cm/s, and 0.63 ± 0.04 in group I, respectively, and 18.32 ± 3.91 cm/s, 6.73 ± 2.04 cm/s, and 0.62 ± 0.11, respectively, in group II. These values showed no statistical difference (*p* > 0.05 in all), and only PS was positively correlated with GFR, demonstrating a moderate correlation (r = 0.48, *p* < 0.05). When analysing the connection between Doppler values and ASL perfusion, a moderate positive correlation was found between PS and ED in comparison with ASL values (r = 0.58, *p* < 0.05, r = 0.56, *p* < 0.05, respectively), while RI showed no significant correlation whatsoever (r = 0.03, *p* = 0.921).

**Figure 6:**
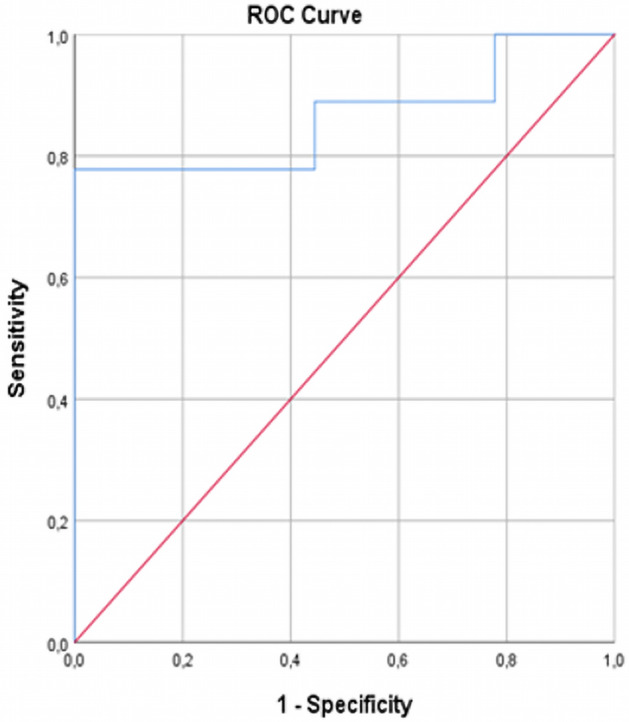
ROC curve of cRBF measured by ASL-MRI for distinguishing allografts with impaired function from allografts with good function.

In the binary logistic regression and ROC curve analyses that took into account all the measured imaging parameters (cRBF, ADC, PS, ED, and RI) in order to predict GFR values in patients, only cRBF showed statistically significant diagnostic efficiency in distinguishing allografts with good or poor function (*p* < 0.05), with a high area under the ROC curve (AUC 0.864, *p* = 0.02), the sensitivity of 78.0%, and specificity of 86.4% (Fig. 6).

## Discussion

In this study, we applied ASL-MRI perfusion, as an absolutely non-invasive tool, to calculate cortical renal blood flow (cRBF) in paediatric and young adult kidney transplant patients in order to discriminate allografts with impaired function.

Overall cortical renal allograft blood flow in our patient group, as measured with ASL, ranged between 85 and 303 mL/100 g/min (mean 183.06 ± 61.66 mL/100 g/min) and it was comparable with previously reported results on transplanted and native kidneys^1,2,5,14–16,20,27–35^. As the mean GFR and mean cRBF value showed no statistically significant difference in comparison to the allograft donor type (*p* = 0.225 and *p* = 0.262, respectively), this allograft characteristic was then considered as uniform in all patients, without statistically significant influence on allograft function. Since all patients were free of acute rejection episodes and were considered clinically stable, we wanted to use cRBF to discriminate the patients with impaired allograft function. Therefore, the patients were divided into two groups according to their calculated GFR value with the threshold of 60 ml/min/1.73 m^2^ determined by the literature. GFR values from two patient groups (with good and impaired function) showed a highly significant statistical difference as expected (*p* < 0.0001).

The main finding in this study was that the mean cortical renal blood flow (cRBF) measured by the ASL-MRI perfusion technique in patients with good allograft function was significantly higher than in patients with impaired function (*p* = 0.0066). Furthermore, renal blood flow measured by ASL-MRI positively correlated with renal function, as determined by the GFR (r = 0.75, *p* < 0.0001), thus leading to the conclusion that cortical perfusion measured by ASL-MRI highly accurately reflected allograft function. Also, the diagnostic efficiency of ASL-MRI in distinguishing allografts with good or impaired function was statistically significant in comparison to other widely used imaging analyses (DWI-MRI, Doppler measurements), with a high area under the ROC curve and high sensitivity and specificity. We would also like to highlight the results of one participant from the group of patients with stable function (participant No 10 in Table 1), who was an outlier concerning the cRBF results from the group. At the time of the study, this participant’s GFR values reflected stable allograft function. However, in the next two months, they showed highly variable GFR values, along with a significant increase in Cystatin C levels (1.31 mg/l) and subsequently a constant GFR decrease over the following 12 months to under 40 ml/min/1.73 m^2^. These clinical facts led to a diagnosis of chronic allograft nephropathy grade 2. In addition, this patient has developed significant portal hypertension. Other patients from this group are still showing stable allograft function.

In this study, we focused on cortical perfusion and haven’t devoted significant attention to medullary perfusion because medullary blood flow is less dependent on hydrostatic pressure and renal vascular resistance in arterial vasculature, given that around 90% of RBF generally exists in cortical microvessels. Additionally, besides targeting immunological mechanisms, the main immunosuppressive therapy (calcineurin inhibitors) principally affects small cortical vessels, reducing cortical perfusion and influencing glomerular filtration. Therefore, the goal of our analysis was to evaluate the relations between the changes in GFR and those in cortical RBF^8,22,24^.

Previous studies have consistently demonstrated that ASL positively correlated with estimated GFR (eGFR) both in native kidneys^2,33,34^ and renal grafts^14,20,31,35^, also reporting that moderately impaired renal function can lead to substantial renal perfusion reduction. Therefore, in patients after kidney transplantation with a decreasing allograft function, ASL-MRI perfusion has the potential to become a useful diagnostic tool for identifying these patients without delay. This fact could also indicate that most invasive procedures for allograft estimation, such as graft biopsy, might not necessarily be performed routinely, especially in paediatric patients, in order to avoid potential complications. Graft biopsy could be reserved for clinically stable patients with detected subclinical impaired function to estimate the underlying pathology and preserve allograft function and longevity.

Our study exhibited similarities to results from previous studies^14,20,31,35^ in which RBF showed a good correlation with the estimated GFR (eGFR). However, most studies evaluating the ASL method published so far have included adult patients with variant MRI approaches and numbers of participants. The patients in our study belonged to the paediatric and young adult population, for which research studies estimating ASL renal allograft perfusion are highly lacking, with a few exceptions, however, involving native kidneys^21^. Also, the number of paediatric participants is significantly limited due to possible later childhood onset of disease or delayed diagnosis of primary illnesses, the more complex preparation of patients for transplantation, certain age, and body weight as possible limitation factors for early childhood transplantation. These could also be potential reasons why studies involving paediatric patients are lacking to a great extent in comparison to studies with adults. In addition, we have also used calculated GFR as a more sensitive and reliable tool reflecting clinical allograft function than eGFR, frequently employed in previous studies.

Conversely, the relatively low number of included patients and variability in the time interval from transplantation to study day in our investigation are potential limitations in tracking the effects of the clinically significant factors influencing allograft perfusion individually or conjointly. Some aspects of population heterogeneity were resolved by recruiting patients who were free of acute rejection episodes. Also, these facts conditioned the exclusion of the blood values of calcineurin inhibitors and their effect on perfusion per se from our analysis, and biopsy results were not available for analysis in a significant number of participants. Some patients were slightly over 18 years old at the moment of data acquisition but had been transplanted as paediatric or very young adult patients; some had significant growth and bone age lag and were going through routine clinical check-ups in our paediatric institution, where they were treated and recruited for the study. Our study population was conditioned by a limited number of patients with clinically stable allograft function at the time of the investigation and in the subsequent 6 months at least, as well as by the general number of accessible patients followed up at our hospital at the time of the study. Also, the lack of acquired T1 maps is a conspicuous limiting factor, as it goes hand-in-hand with ASL. We are aware that T1 maps are crucial in tissue characterisation, especially in tracking longstanding chronic allograft rejection. However, during our study, we were unable to collect them from all participants and, therefore, used well-known literature values^30,31^. We assume that if we had measured the T1 value, the difference in perfusion could have been even more prominent, as it is an exponent in the equation. We would have expected to observe a significant difference in T1 value between the groups (with higher T1 corresponding to lower GFR) since T1 and cRBF should show a negative correlation.

However, the main point of our study was the evaluation of ASL-MRI as a potentially novel, completely non-invasive method of renal allograft perfusion assessment for discriminating allografts with impaired function and its comparison to some standard clinical biomarkers. Limitations of ASL as a method consider its relatively low signal-to-noise ratio and long acquisition time, the problems that can be highlighted in the paediatric population due to lack of cooperation. Its routine clinical application demands additional and extensive investigations with various approaches and study populations. We believe that more time points of measuring ASL-MRI perfusion in the early posttransplant period and the subsequent few years are necessary to track all the possible complications that lead to impaired allograft function at any posttransplant time, as well as the effects of immunosuppressive therapy, especially calcineurin inhibitors. All these facts imply that for more detailed analysis, especially for determining threshold values of cRBF for good or impaired allograft function, larger number of patients, paediatric and adult, and multicentric studies are imperative.

We know that the biexponential IVIM and ASL model is even more sensitive in perfusion evaluation^31,35^. However, the reason we chose to perform a standard DWI is that, first, we wanted to perform a routine sequence used in standard clinical protocols for evaluating kidneys (either native or allografts) and see how powerful it would be in discriminating patients with good or impaired allograft function. Secondly, IVIM lasts much longer than the monoexponential model, which is simpler, more robust and requires only a moderate signal-to-noise ratio, especially in small subjects, such as paediatric patients. Our ADC values revealed no difference between the two patient groups, suggesting that conventional DWI is not a reliable tool in analysing allografts with clinically stable function. On the other hand, DWI has a potential role in diagnosing acute or chronic complications, such as acute ischaemia or infection, which lead to largely impaired allograft anatomy or function. Yet, for a minutious graft analysis, different DWI approaches involving the comparison to allograft biopsy and a greater number of patients are necessary^35^.

In our study, Doppler measurements were confirmed to be insensitive and nonspecific in tracking allograft function. Peak-systolic velocity (PS) showed a moderate correlation to GFR and, as an isolated parameter, has no clinical significance. Conversely, the clinically more important resistive index showed no correlation to GFR or cRBF in any group, indicating that the widely used ultrasound measurements cannot reliably discriminate patients with poor allograft function.

## Conclusion

In conclusion, our results suggest the cortical renal allograft blood flow as measured by the ASL-MRI perfusion technique differs between patients with good and impaired allograft function and correlates significantly with allograft function, allowing for its non-invasive assessment.

## Data Availability

The datasets generated and/or analysed during the current study are available from the corresponding author on reasonable request.
